# Can Gender and Age Impact on Response Pattern of Depressive Symptoms Among College Students? A Differential Item Functioning Analysis

**DOI:** 10.3389/fpsyt.2019.00050

**Published:** 2019-02-12

**Authors:** Antonio Reis de Sá Junior, Graziela Liebel, Arthur Guerra de Andrade, Laura Helena Andrade, Clarice Gorenstein, Yuan-Pang Wang

**Affiliations:** ^1^Department of Health Sciences, Federal University of Santa Catarina, Araranguá, Brazil; ^2^Institute and Department of Psychiatry (LIM-23), University of São Paulo Medical School, São Paulo, Brazil; ^3^Department of Collective Health, Federal University of Juiz de Fora, Juiz de Fora, Brazil; ^4^Department of Neuroscience, Medical School, ABC Foundation, Santo André, Brazil; ^5^Department of Pharmacology, Institute of Biomedical Sciences, University of São Paulo, São Paulo, Brazil

**Keywords:** depression, gender, age, college students, Beck Depression Inventory, item response theory, differential item functioning

## Abstract

**Background:** Self-reported depressive complaints among college students might indicate different degrees of severity of depressive states. Through the framework of item response theory, we aim to describe the pattern of responses to items of the Beck Depression Inventory-II (BDI-II), in terms of endorsement probability and discrimination along the continuum of depression. Potential differential item functioning of the scale items of the BDI-II is investigated, by gender and age, to compare across sub-groups of students.

**Methods:** The 21-item BDI-II was cross-sectionally administered to a representative sample of 12,677 Brazilian college students. Reliability was evaluated based on Cronbach's alpha coefficient. Severity (*b*_*i*_) and discrimination (*a*) parameters of each BDI-II items were calculated through the graded response model. The influence of gender and age were tested for differential item functioning (DIF) within the item response theory-based approach.

**Results:** The BDI-II presented good reliability (α = 0.91). Women and younger students significantly presented a higher likelihood of depression (cut-off > 13) than men and older counterparts. In general, participants endorsed more easily cognitive-somatic items than affective items of the scale. “Guilty feelings,” “suicidal thoughts,” and “loss of interest in sex” were the items that most likely indicated depression severity (*b* ≥ 3.60). However, all BDI-II items showed moderate-to-high discrimination (*a* ≥ 1.32) for depressive state. While two items were flagged for DIF, “crying” and “loss of interest in sex,” respectively for gender and age, the global weight of these items on the total score was negligible.

**Conclusions:** Although respondents' gender and age might present influence on response pattern of depressive symptoms, the measures of self-reported symptoms have not inflated severity scores. These findings provide further support to the validity of using BDI-II for assessing depression in academic contexts and highlight the value of considering gender- and age-related common symptoms of depression.

## Introduction

Depression is among the most prevalent psychiatric disorders worldwide and has been associated with some demographic determinants, including gender and age. The prevalence of depression is higher among women when compared to men, with evident sex ratio of 2:1 of diagnosed depression after adolescence (1, 2). The underlying mechanism of this gender difference is not fully understood. Possibly, the reported variation is due to the joint effect of biological factors, including genetic, physiologic, and endocrine determinants, and social or demographic factors, such as gender-specific roles and coping strategies (3).

Depression scales are commonly assumed to measure the same attributes for both males and females. Insufficient evidence of measurement bias compromise the conclusions based on gender group. Studies regarding gender differences in depression usually depend on the mean comparisons estimated by depression scales. However, the mean differences in depression can be attributed to a true difference, measurement bias, or a combination of both. It appears that few studies have investigated the gender-related measurement bias of the BDI-II score when making a comparison of gender (4).

Regarding age, late adolescence is a transitional period during which young adults experience stressful socio-biological transformations. Notably, psychiatric disorders are common among college-aged individuals (5). The first onset of depression in adulthood, particularly among college students around their 20s, appears to be higher than in those non-college adults in their 30s or 40s (6–8). Frequently, undergraduate students have to cope with part-time work and family demands in addition to academic requirements. Also, searching for a formal job in the working world is an additional distress for many students before completing graduation. Thus, the observed gender and age-related differences of emotional symptoms among college students may indicate which group of individuals would most benefit from prevention and timely management of depression.

Traditionally, the diagnosis of depression is reached through standardized interviews and complemented with assessment scales. Most instruments for symptomatic assessment of depression are built on the ground of classical test theory (CTT), where the item scores are summed to indicate severity and a same weight is attributed to each scale item. In this classical approach, the probability of item endorsement changes in accordance with the characteristics of participants that take a specific test, obstructing the comparability of results (9, 10).

For instance, the 21-item Beck Depression Inventory-II (BDI-II) is one of the most commonly used self-report measures of severity of depressive symptoms (11). The popularity of the BDI-II has been attributed to its rigorous psychometric evaluation (12). The BDI-II may be scored either by summing a number of equal-weighted items or fitting a measurement latent model, yielding a cumulative score as a continuous variable. Nevertheless, the classical method cannot provide direct guidance regarding the ability of BDI-II to assess depressive symptomatology at various points of the severity continuum of depressive syndrome. Individuals reporting suicidal thoughts may indicate more severe states of depression than those with sleeplessness. Common symptoms in academic environments, like fatigue or loss of energy, changes in sleep and appetite, might be misinterpreted as reliable mood-related indicators of severity (13). In medical contexts, while some individuals report more somatic symptoms, others may predominantly complain of cognitive-affective symptoms (14). Item analysis of the scale (15) may examine the confounding effect of the somatic symptoms on the measurement of depressive symptoms in people diagnosed with a chronic disease (16, 17).

In recent decades, research demonstrated that diagnostic and screening instruments could benefit from refined statistical approaches, such as the item response theory (IRT) models (10). This family of psychometric methods is considered an appropriate and robust approach to address the measurement properties of rating scales, for example, those built for measuring depression (15). The basic assumption in IRT asserts that the latent trait θ (theta, in Greek) of a participant is independent of the content of a test (18). The IRT model provides information on the relationship between responses to a set of items (probability of endorsement/non-endorsement of depressive symptoms, or parameter *b*_i_) and the value of a person's latent trait (difficulty or the severity of depression). Each item can be associated with a discrimination parameter (*a*_i_), which denotes the extent to which responses on the item reliably indicate differences between persons' overall severity scores. High item discrimination suggests the symptom has a stronger association with the underlying depression dimension θ (19–21).

The IRT can examine item bias of a psychometric tool across diverse population groups. The differential item functioning (DIF) tests how similar is the between-group item perform (22). The measurement equivalence is a necessary condition for meaningful comparison among different sub-groups. The variety of existing depression scales, the methodological differences of assessment, and the diversity of group variables examined for DIF across the reviewed studies make the synthesis of findings on depression a challenging task (13, 19, 20, 23).

Hypothetical DIF can be generated based on the fact that women present higher scores on self-reported depression measures than men (24, 25) and there are also age-related differences in the manifestation of depressive symptoms (26). Consequently, some symptoms would reflect a more severe depressive state than other symptoms among college students. As the BDI-II is intended to be a clinically useful measure of depression, it is expected that some items might discriminate between individuals with high and low levels of depression. Therefore, group-level differences are expected, since the assessment of rates or severity of depression might reflect DIF with respect to expression of symptoms by gender or age sub-groups.

In the current study, the item performance of the BDI-II was examined in a representative sample of Brazilian college students. The underlying construct considered herein is “self-reported depression.” The aim of the current paper was to present item-level psychometric findings of the BDI-II for college students, in the form of IRT parameters and DIF analysis regarding gender and age. Strengths and limitations of results found in item analyses are discussed.

## Materials and Methods

### Ethics Statement

The Ethics Committee for the Analysis of Research Projects at the University of São Paulo Medical School approved the present study. All participants provided written informed consent prior to participating in the data collection.

### Sampling Frame

The target population was students enrolled in the “I Nationwide survey on the use of alcohol, tobacco, and other drugs among college students in the 27 Brazilian state capitals” (27). This sample was recruited through two-stage cluster sampling. The first stage involved higher education institutions (HEIs) that deliver undergraduate courses in each of 27 state capitals, in accordance with the school list provided by the Brazilian Ministry of Education. Eligible HEIs were randomly selected from the list of ordered institutions, by the means of the probability proportional to size (PPS) of the number of enrolled students in each HEI. The stratification was based on the two variables: the location of capital and the type of institution funding (either private or public), totaling 54 levels. Of the selected 114 colleges and universities, 100 HEIs (87.7%) agreed to participate after institutional invitation.

In the second stage, the class of students was considered the primary sampling unit (PSU) within each selected HEI, for the sake of feasible fieldwork. Each element of the class was defined as a group of students attending a given course. The number of classes was proportional to the total number of students in the institution, totaling 654 classes or 70.6% of selected PSU. All students of the selected classes were invited to volunteer for the study. The participants' response rate was 95.6% for the college students who were in classes at the time of the survey (27).

### Participants

The initial sample of the survey was 12,721 college students. Of these, 10 respondents were excluded because they claimed to have used the dummy drug “relevin” and 34 did not report gender. Hence, a final sample of 12,677 valid questionnaires (99.7%) was considered for analysis.

All students completed a structured, anonymous, self-administered instrument consisting of 98 closed questions on the use of substances, wherein the 21-item Beck Depression Inventory-II (BDI-II) was included. The demographic characteristics of the sample were 5,692 men (44.9%) and 6,985 women (55.1%), with the mean age of 23.9 years (*SD* = 6.9; range 16–84 years). On average, the mean age of women was similar to men (*M* = 23.8 vs. 24.0, *p* = 0.15). Because the distribution of age was left-skewed, with the greatest majority of participants aged in late adolescence and 20s, the age group was dichotomized as younger participants (16–30 years old; *n* = 10,887, *M*= 21.7, and *SD* = 3.1) and older participants (31–84 years old; *n* = 1,790, *M*= 38.7, and *SD* = 7.0). Older participants were divided as follows: 31–40 years old 66.8%; 41–50 years old 26%; 51–60 years old 6.6%; 61–70 years old 0.4%, and 71–84 years old 0.2%.

Additionally, the self-reported skin color—as the proxy for race/ethnicity in Brazil—showed that most of the sample consisted of white students (55.5%), followed by black (6.8%), mulatto (29.8%), Asian (2.9%), Indian (0.8%), other mixed colors (3.0%), and 1.3% no reply. When asked about “What is your present religion?” the answers were: 16.3% with no religion, 53.0% Catholic, 18.4% Evangelical or Protestant, 11.8% others religions, and 0.5% no replay. Marital status had the following rates: 80.5% single, 16.9% married, 1.7% divorced, 0.1% widowed, and 0.7% no reply.

### The Beck Depression Inventory-II (BDI-II)

The BDI-II is a 21-item self-report questionnaire for assessing the severity of depressive symptoms, which items consist of four ordinal categories (from 1 through 4). Absence (or “as usual”) of depressive symptom in each item is scored as “0” and an increasing presence of symptoms is endorsed between 1 and 3. High scores, either in an item or in the scale, indicates more intense symptom severity. Possible total score ranges from 0 to 63. The scale content reflects the cognitive, affective, somatic, and vegetative symptoms of depression (11).

The timeframe of the BDI-II covers the 2 weeks requirement to meet the DSM-IV criteria for major depressive disorder (28). Substantial evidence of reliability has been reported in different language versions of the BDI-II (11), including the Brazilian-Portuguese (29). The BDI-II has been validated for psychiatric and medical patients, students, and adolescent samples (11, 12, 30). Considerable evidence of construct validity has been demonstrated in college student samples (31–33).

### Statistical Analyses

First, the Cronbach's alpha (α) coefficient of internal consistency was calculated. Characteristics of BDI-II data dispersion and homogeneity were explored for each scale item and a total score: mean (*M*), standard deviation (SD), Pearson's coefficient of variation (CV) and Cronbach's α if the item is deleted (item-total analysis).

There are two assumptions for IRT: unidimensionality (i.e., there is only one underlying factor within the data) and local independence (items should not be correlated when the shared variance of the latent trait is removed). Both assumptions were tested through confirmatory factor analyses (CFA), to examine the covariance structure of the scale. To evaluate model fits, one absolute close-fit index, that is, root mean squared error of approximation (RMSEA) and two incremental close-fit indexes, that is, comparative fit index (CFI) and Tucker-Lewis index (TLI) were used (34). The following cut-off points were considered as indicative of an adequate model fit: RMSEA < 0.06, CFI and TLI ≥ 0.95 (35, 36). The results of CFA approximately fitted data to unidimensional model: $\chi_{\left(210\right)}^{2}$ = 9054.21, *p* < 0.001, CFI = 0.95, TLI = 0.94, and RMSEA = 0.06. The significance of χ^2^ test was magnified due to the large sample size. These indicators favored the use of IRT model (37).

For a polytomic ordinal scale, the graded response model (38) was adequate to describe the relationship between the observed responses to the BDI-II and the underlying latent trait θ (theta), as the proxy of depression severity. This model yields discrimination (*a*) and severity or threshold (*b*_*i*_) parameters for each criterion. Scale items (i) with a large slope (a) provide more information or discrimination for the depressive symptoms. The item difficulty (bi) is distributed along a latent dimension θ (theta) to reflect the trait of severity in relation to absence or presence of the pathological condition. In health sciences, the b score of a given scale item (i) used to locate in the right side of the latent variable θ to denote more severe manifestation of the target illness (21). For interpretation, Baker (21) suggests that discrimination parameter values *a* ranging from 0.01 to 0.24 are considered very low, 0.25–0.64 low, 0.65–1.34 moderate, 1.35–1.69 high, and more than 1.7 very high.

Detection of differential item functioning (DIF) was performed in the framework of iterative hybrid ordinal logistic regression (OLR) (39). This state-of-the-art approach incorporates IRT-derived trait scores θ as the estimator of the “ability” to score an item, rather than the observed BDI-II domain cumulative score as in OLR. The generic term “ability” can be used hereby to represent the trait score as measured by the test, either as the observed sum score on the BDI-II or the latent variable (the *b*_*i*_ parameter). The dependent variable of regression models was the item response (0–3) and the independent variable was the IRT-derived trait score, i.e., the level of depression on the BDI-II scale.

Analyses were conducted to identify biased items showing gender and age difference. Respondents' gender was dichotomized as men or women and the age as younger or older participants. For each item, a null model and three nested models were estimated hierarchically with additional explanatory variables. In the null model 0, only the intercept was considered in the equation. In the baseline model 1, the trait score θ was used as the predictor of the item response in addition to the intercept. In model 2, both trait score θ and gender/age predicted the item response (DIF). In model 3, an interaction term was included between the trait score θ and gender/age.

Comparisons were performed to assess the magnitude and the type of DIF for flagged items, by the means of likelihood-ratio χ^2^ and pseudo *R*^2^ statistics for comparing three nested logistic regression models. First, if the difference between models 1 vs. 3 showed an *R*^2^ difference > 0.02, the flagged item was considered to show DIF (39). Second, if the difference between models 2 vs. 3 showed an *R*^2^ difference > 0.02, then the item was considered to show “non-uniform DIF” because the effect varies conditional to the trait level. Third, if the difference between models 1 vs. 2 showed an *R*^2^ difference > 0.02, the item was considered to show “uniform DIF,” because the effect was constant. Final comparison tested whether DIF was uniform, i.e., consistent impact across endorsement probability or non-uniform, i.e., the probability that varied according to levels of endorsement.

We plotted the item characteristic curve (ICC) of the DIF items for both genders and age to inspect the direction of the difference. Finally, the test characteristic curve (TCC) provides insight into the actual impact of DIF items on the total score.

While descriptive analyses and CFA were computed through Mplus 6.12 (www.statmodel.com), the estimates of the IRT model and DIF analyses were calculated through R 3.5.0 (cran.r-project.org). The *lordif* library was implemented to compute the regression models. The level of significance was set as 0.05 for two-tailed tests.

## Results

### Reliability and Descriptive Analysis

Cronbach's α reliability was 0.91 and did not differ between genders (men = 0.90; women = 0.91) or age (younger and older participants = 0.91). Descriptive statistics of responses obtained from 12,677 respondents for BDI-II are shown in Table 1, the mean total score was 6.99 (SD 7.56, CV 1.08). The average BDI-II total score between women (*M* = 7.85, *SD* = 8.08) and men (*M* = 5.92, *SD* = 6.72) or younger participants (*M* = 7.17, *SD* = 7.64) and older participants (*M* = 5.83, *SD* = 6.91) were significantly different (*p* < 0.001). Also, using the recommended cut-off score of 13 (11), the proportion of probable depression was significantly different in relation to gender (men = 14.2%; women = 22.1%; *p* < 0.001) and age (younger participants = 19.2%; older participants = 14.7%; *p* < 0.001).

**Table 1:** Psychometric characteristics of the Beck Depression Inventory-II for college students (*N* = 12,677).

**Item**	***M***	***SD***	**CV**	**α**
1- Sadness	0.21	0.46	2.05	0.90
2- Pessimism	0.22	0.48	2.05	0.90
3- Past failure	0.23	0.57	2.25	0.90
4- Loss of pleasure	0.26	0.52	1.83	0.90
5- Guilty feelings	0.33	0.53	1.53	0.90
6- Punishment feelings	0.22	0.62	2.33	0.90
7- Self-dislike	0.24	0.63	2.50	0.90
8- Self-criticalness	0.56	0.72	1.14	0.90
9- Suicidal thoughts	0.08	0.32	3.57	0.90
10- Crying	0.36	0.77	1.91	0.90
11- Agitation	0.35	0.61	1.55	0.90
12- Loss of interest	0.26	0.52	1.83	0.90
13- Indecisiveness	0.41	0.78	1.61	0.90
14- Worthlessness	0.17	0.52	2.67	0.90
15- Loss of energy	0.44	0.62	1.29	0.90
16- Changes in sleep	0.66	0.75	0.98	0.90
17- Irritability	0.37	0.61	1.48	0.90
18- Changes in appetite	0.48	0.69	1.29	0.90
19- Concentration difficulty	0.46	0.72	1.38	0.90
20- Tiredness or fatigue	0.51	0.70	1.20	0.90
21- Loss of interest in sex	0.15	0.45	2.75	0.90
Total	6.99	7.56	1.08	0.91

*M, Mean; SD, Standard deviation; CV, Pearson's coefficient of variation = SD/M; α, Cronbach's alpha coefficient of internal consistency if the item is deleted*.

Table 2 presents the frequency distributions for five arbitrarily selected items: “sadness” (#1), “guilty feelings” (#5), “crying” (#10), “loss of energy” (#15) and “loss of interest in sex” (#21). The frequency distributions for 21 items are presented in a table as a Supplementary File. Inspecting the table, the great majority of respondents endorsed the category 1 of items, which denotes “as usual” or “unchanged” manifestation of depressive symptoms. The symptomatic categories of 2, 3, and 4 were endorsed in decreasing frequency, intensity or distress, to indicate the severity of depressive symptoms. As expected for this non-clinical sample of analogous students, the more severe the symptom, the less it was chosen by the respondents. Examining the columns for gender and age groups, a considerable difference was observed for specific items. For example, women endorsed more “sadness,” “crying,” and “loss of energy” than men did. On the other hand, while “crying” was more likely endorsed by younger participants, “loss of interest in sex” was more reported by older counterparts.

**Table 2:** BDI-II response proportion (%) for responses categories on selected items.

**Item**	**Overall *N* = 12,677**	**Men *n* = 5,692**	**Women *n* = 6,985**	**Younger *n* = 10,887**	**Older *n* = 1,790**
**1. SADNESS**					
Category 1	81.3	85.7	77.6	80.3	86.9
Category 2	17.5	13.2	20.9	18.3	12.3
Category 3	0.7	0.5	0.8	0.8	0.3
Category 4	0.6	0.5	0.6	0.6	0.5
**5. GUILTY FEELINGS**					
Category 1	69.9	72.4	67.9	69.3	73.9
Category 2	28.1	25.9	30.0	28.7	24.8
Category 3	1.4	1.3	1.5	1.4	1.1
Category 4	0.6	0.5	0.6	0.6	0.2
**10. CRYING**					
Category 1	77.6	88.4	68.9	76.7	83.5
Category 2	12.1	6.4	16.7	12.6	8.8
Category 3	6.5	0.8	11.2	7.0	3.9
Category 4	3.8	4.5	3.1	3.7	3.8
**15. LOSS OF ENERGY**					
Category 1	61.6	66.3	57.7	61.9	59.4
Category 2	33.2	30.1	35.8	32.6	36.4
Category 3	4.5	3.2	5.7	0.47	3.9
Category 4	0.7	0.5	0.9	0.8	0.3
**21. LOSS OF INTEREST IN SEX**					
Category 1	88.0	91.9	84.8	89.6	78.3
Category 2	9.4	6.9	11.4	8.0	17.7
Category 3	2.0	0.9	2.9	1.9	3.0
Category 4	0.6	0.3	0.9	0.5	1.1

*In general, category 1 indicates “as usual”; category 2 “more than usual”; category 3 “all the time”; and category 4 “extreme distress.” Although the item wording may vary according to the depressive symptom, the categories are displayed in increasing level of severity*.

The proportion of responses to each item was not uniform, which variation suggests a differential probability of endorsement rate according to discrimination and difficulty level of an item in the BDI-II. Moreover, these results indicate noteworthy differences in the gender- and age-related response pattern of self-reported depressive symptoms. Item analysis and differential item functioning (DIF) are explored below to understand their impact on the between-group difference of depression, both by the mean score and by the cut-off threshold.

### Item Analysis

Table 3 shows discrimination (*a*) and severity threshold (*b*_i_) estimates of depressive symptoms for all 21 items. The discrimination (*a*) parameters ranged from 1.32 (#21 “loss of interest in sex”) to 3.31 (#14 “worthlessness”), suggesting that all scale items presented moderate-to-high discriminatory characteristics for the latent trait of “self-reported depression” (θ).

**Table 3:** Estimated graded response model (GRM) parameters of the items of the BDI-II (*N* = 12,677).

**Item**	***a***	**(*SE*)**	***b*_**1**_**	**(*SE*)**	***b*_**2**_**	**(*SE*)**	***b*_**3**_**	**(*SE*)**
1. Sadness	2.38	(0.06)	1.10	(0.05)	2.75	(0.12)	3.13	(0.15)
2. Pessimism	2.20	(0.05)	1.12	(0.05)	2.57	(0.09)	3.35	(0.16)
3. Past failure	2.04	(0.05)	1.32	(0.05)	1.98	(0.07)	3.41	(0.14)
4. Loss of pleasure	2.27	(0.05)	0.95	(0.05)	2.34	(0.08)	3.25	(0.15)
5. Guilty feelings	1.72	(0.04)	0.75	(0.03)	2.96	(0.08)	3.78	(0.13)
6. Punishment feelings	1.74	(0.05)	1.46	(0.05)	2.39	(0.07)	2.63	(0.07)
7. Self-dislike	2.86	(0.07)	1.24	(0.07)	1.60	(0.09)	2.54	(0.14)
8. Self-criticalness	1.62	(0.03)	0.25	(0.03)	1.78	(0.04)	3.24	(0.08)
9. Suicidal thoughts	2.12	(0.07)	1.89	(0.08)	3.22	(0.15)	3.60	(0.19)
10. Crying	1.52	(0.04)	1.16	(0.03)	1.93	(0.04)	2.73	(0.06)
11. Agitation	1.59	(0.04)	0.83	(0.03)	2.90	(0.06)	3.19	(0.08)
12. Loss of interest	2.73	(0.06)	0.89	(0.05)	2.26	(0.11)	2.87	(0.15)
13. Indecisiveness	2.03	(0.04)	0.78	(0.04)	1.88	(0.06)	2.12	(0.06)
14. Worthlessness	3.31	(0.09)	1.31	(0.10)	1.80	(0.12)	2.72	(0.20)
15. Loss of energy	2.35	(0.05)	0.40	(0.04)	1.96	(0.07)	3.05	(0.13)
16. Changes in sleep	1.58	(0.03)	0.03	(0.03)	1.69	(0.04)	3.04	(0.07)
17. Irritability	2.13	(0.04)	0.65	(0.04)	2.10	(0.07)	2.93	(0.11)
18. Changes in appetite	1.40	(0.03)	0.48	(0.03)	2.31	(0.04)	3.28	(0.07)
19. Concentration difficulty	2.07	(0.04)	0.57	(0.03)	1.60	(0.05)	2.84	(0.09)
20. Tiredness or fatigue	2.24	(0.04)	0.32	(0.03)	1.75	(0.06)	2.52	(0.09)
21. Loss of interest in sex	1.32	(0.04)	1.91	(0.04)	3.26	(0.07)	4.44	(0.12)

*a is the discrimination parameter; the b_i_ are location parameters; SE is standard error of the parameter estimate*.

All severity or difficulty parameters (*b*_i_) presented positive value, on the right side of the θ. The first (*b*_1_), second (*b*_2_), and third (*b*_3_) threshold parameters ranged from 0.03 to 1.91, 1.60 to 3.26, and 2.12 to 4.44 respectively. The ascending sequence of the thresholds *b*_1_, *b*_2_, and *b*_3_ confirmed the appropriate direction of response options. The BDI-II item location parameters (*b*_i_ threshold) were fairly consistent over an increasing gradient of the score in terms of latent trait θ.

For the threshold parameter *b*_1_, the easiest BDI-II items were #16 “changes in sleep,” #8 “self-criticalness,” and #20 “tiredness of fatigue.” The more severe items for *b*_1_ were #21 “loss of interest in sex,” #9 “suicidal thoughts,” and #6 “punishment feelings.” In the same direction, for the threshold parameter *b*_3_, the easiest BDI-II items were #13 “indecisiveness,” #20 “tiredness of fatigue,” and #7 “self-dislike.” The more severe items on *b*_3_ were #21 “loss of interest in sex,” #5 “guilty feelings,” and #9 “suicidal thoughts.” Worth noting, the small value of standard error (SE) of all parameters indicated the good precision of the estimates in this large sample. Furthermore, the higher the endorsement rate of a given item, the smaller or more precise was the value of the SE.

Because the threshold parameters provided uneven information of score threshold distribution, we examined the pattern of response for gender and age sub-groups, by the means of DIF analysis.

### Differential Item Functioning

Regarding gender-related DIF, the *lordif* method detected the item #10 (“crying”) as a problematic element; with women more likely endorsed this symptom than men. For age-related DIF, item #21 (“loss of interest in sex”) also indicated a potential difference in the response pattern; with younger participant less likely endorsed this symptom than older counterparts.

In gender and age analyses implemented by ordinal logistic regression (OLR), the pseudo *R*^2^ differences between models 1 vs. 3 were, respectively 0.024 and 0.027, which indicated overall DIF. On the other hand, the pseudo *R*^2^ differences between models 2 vs. 3 were < 0.001 for both items and indicated the presence of uniform DIF. The complete table of the OLR models for all BDI-II items is not shown, but it is available upon request to the authors.

**Figure 1:**
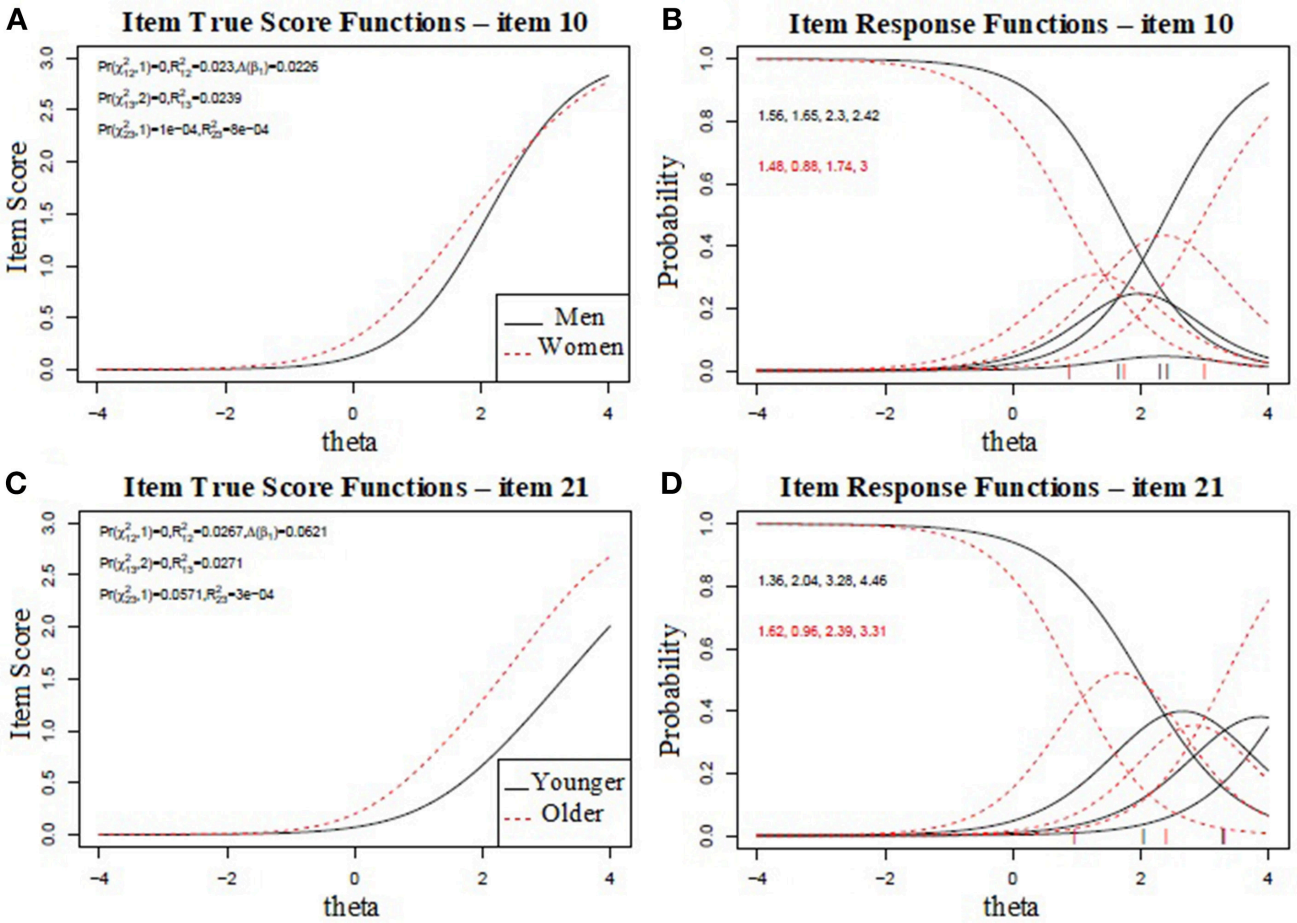
The first **(A)** and third **(C)** plots represent the true score (item response theory score) of the college student sample on differential item functioning (DIF), for item #10 and item #21. The second **(B)** and fourth **(D)** plots show the item characteristic curves for items #10 and #21. The curves show the probability of endorsing a particular item.

The item characteristic curve (ICC) showed that participants from women and older sub-groups present higher likelihood of endorsing items #10 and 21 than men and younger participants, respectively (Figure 1). The response curves for both items are displayed in an ordered sequence along the latent trait θ. Dotted curves indicate that, given a similar latent trait level, the women and older participants had a higher chance to endorse these items than men and younger counterparts. From a further investigation of the ICC for male respondents, it is indicated that the men sub-group presented a substantial overlap for b_1_ and b_2_ threshold on item #10. This suggests that the threshold for endorsing “crying” in men's sub-group needs be re-calibrated.

**Figure 2:**
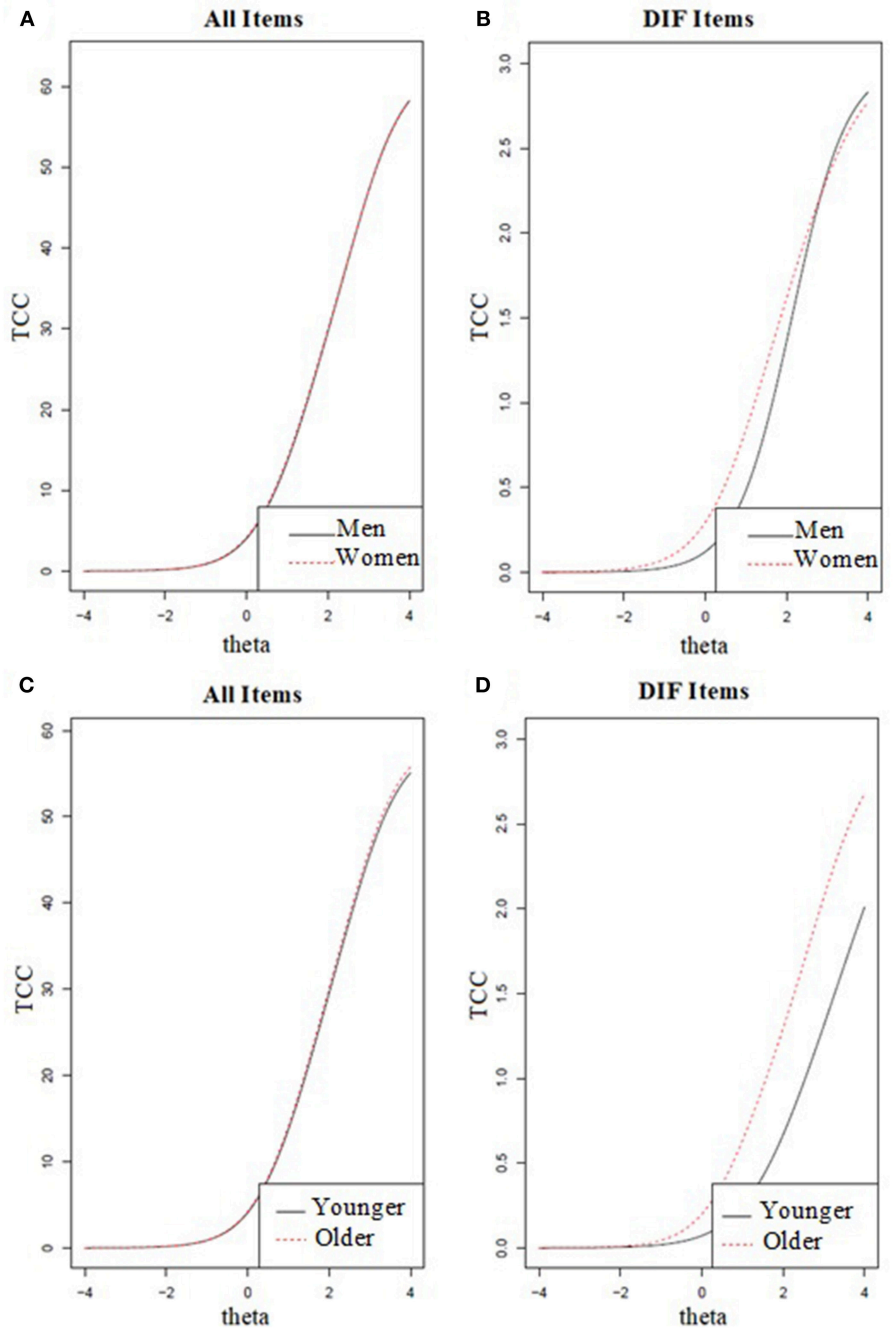
Overall impact of differential item functioning (DIF) for gender and age on the test characteristic curves (TCC). The first **(A)** and third **(C)** plots display the TCC of all items and the second **(B)** and fourth **(D)** plots display the TCC of specific DIF items (i.e., item #10 for gender and item #21 for age).

Figure 2 displays the test characteristic curve (TCC) for all items and gender/age sub-groups. The figures show the overall impact of DIF for gender and age on TCCs for all 21 BDI items (ignoring DIF) and the individual impact of DIF-anchored item #10 and #21 on TCC. Inspecting the plots, it is clear that there was no substantial influence of DIF in items #10 and #21 on the total domain score, because of the similar slopes and overlap in scorings between the two sub-samples. These findings indicated a minimal impact of DIF by gender or age, even though the expected total score was higher for women than men and older adults than younger adults. To summarize, although items #10 and #21 showed DIF, these items only had a negligible impact on the total score.

## Discussion

The present study has brought remarkable information on gender and age bias on the response pattern of depressive symptoms in a large representative sample of college students. According to classical test theory framework, the BDI-II has shown sufficient internal consistency to capture the underlying construct of “major depression.” While all 21 items have proved to discriminate well the latent trait of “depression,” some items were examined with respect to respondent's characteristics. The major findings indicated that self-reported symptoms of “crying” and “loss of interest in sex” might modify the estimate of depression. Thus, this item-wise psychometric study provided an opportunity to compare the measurement of depression across different groups.

Regarding discrimination power (the *a* parameter), the item response analysis has indicated that all BDI-II items have presented at least moderate ability (21) to discriminate the latent trait along the severity continuum of “depression.” In comparison, the somatic items (“loss of interest in sex” and “changes in appetite”) were less informative than affective items (“worthlessness” and “self-dislike”). Therefore, all BDI-II items were suitable to depict depression among college students.

Regarding the item difficulty (the *b*_i_ parameter), the most endorsed items were the least severe ones (13, 40, 41). The excess of sub-threshold depressive symptoms (total score < 13) in this student sample suggested that the BDI-II items were hard to endorse but capable of detecting depressive symptoms within a wide range of severity. If a scale purposing to measure depression were composed only of items measuring mild depressive states, the instrument would have great trouble for distinguishing between moderate and severe cases of depression: both would be characterized by high scores on all items. On the other hand, if a scale's items easily detect somatic symptoms of depression, cognitive-affective phenotypes of depression might remain unnoticed (42–44). In general, the BDI-II items cover either somatic types or cognitive-affective types of depression in a single dimension of severity.

In theory, individuals with a given depression severity level also should have the same likelihood of scoring a given item response. When men and women or younger and older adults with the same level of depression severity systematically respond to an item in different ways, the item is malfunctioning for different groups of respondents (45). Therefore, the DIF analysis examines whether or not the likelihood of item endorsement is equal across gender and age sub-groups that are matched on the state or trait measured (e.g., depression). When DIF is absent across 21 items of the BDI-II, meaningful comparison of the distribution of latent scores across heterogeneous populations might be accepted. If the DIF is present and ignored, estimates of prevalence or severity might be biased.

In the present study, item “crying” (#10) exhibited significant gender DIF and item “loss of interest in sex” (#21) exhibited significant age DIF. Also, these items were found to show uniform DIF based on the pseudo *R*^2^ change. A high conditional endorsement was observed for women on #10 and older participants on #21. The direction of item malfunctioning indicated that a sample with more women than men might overestimate the prevalence and severity of depression due to #10. Conversely, a younger-participant sample might under-estimate cases of depression due to #21.

Episodes of crying can be viewed as an adaptive coping response to stress, but it should not be automatically interpreted as a sign or symptom of depression. In the present study, crying spells were reported by almost one in four respondents. This behavior has moderately discriminated the severity of depression and displayed a modest threshold to be expressed by the students. However, there is no rigorous empirical foundation on the relationship between crying and depression (46, 47). Most assessment instruments and diagnostic systems for mood disorders are unreliable in how they handle crying as a symptom (48). For a higher diagnostic accuracy, it is important that the criteria and instruments used to assess depression adequately reflect female and male common depressive symptoms.

Most observed among women, the item “crying” as a gender-bounded emotional response has been claimed in most studies with depressive scales to demonstrate DIF (19, 20, 49–54). Although significant gender DIF also was found in our investigation for “crying,” the meaning of this symptom to the assessment of depression remains a matter of debate. We have shown that the thresholds for “crying” seem to be quite different among female and male adults. While female students were more easily tearful than male colleagues, the overall score did not impact on the severity assessment of depression. Possibly, our gender-balanced large sample might have concealed substantial evidence of this difference. In order to prevent over- or under-estimate of depression, researchers must beware when interpreting the results of studies with the dominance of female participants, and vice-versa.

Sexuality, both libido and sexual functioning, is viewed as an important component of emotional and physical intimacy that most people experience throughout their lives. Depressive symptoms have been associated with low sexual desire in 50–60% of untreated patients with diagnosed depression (55). For instance, Toosi et al. (56) showed lower endorsement of the “loss of interest in sex” were depressive symptoms associated with aging. Many medical diseases become increasingly prevalent with aging, blurring boundaries between depression symptoms and physical complaints. As a consequence, depression may remain under-detected because both types of symptoms may co-occur and interact as physical-mental comorbidities (57, 58). Other sources of confusion include unwillingness among elderly individuals to not disclosure their sexual life, stigma to psychiatric labels (59), and the belief that depression is simply expected when they get older (60).

When measured as a continuous variable through sum scoring, factor analysis or item response model, levels of depression generally increase with age (61–63). However, many health care professionals are reluctant to address this issue during a routine encounter, without an active inquiry of embarrassing topics (64). In this way, it is not surprising that sexual dysfunction remains an under-recognized problem in clinical practice. Likewise to “crying,” the impact of “loss of interest in sex” should be understood in light of the age distribution of the sample.

Differences in the measurement of variables such as gender or age might partially explain the discrepancy between previous prevalence studies using symptom scales. Taking advantage of recent psychometric advance of IRT, the detection of DIF for a specific variable did not inevitably indicate that a given item is biasing the reported estimates. Measurement bias is typically identified after considerable empirical evidence has been gathered and reviewed by experts to determine the potential impact of the DIF (23). In the current study, it was suggested that the BDI-II appears to perform similarly for men and women or for younger and older adults. Therefore, gender or age-based differences in prevalence estimates of depressive symptoms are less important than previously supposed. Future research using representative epidemiological datasets is warranted to replicate the key findings reported in the current study on the effects of gender or age in depressive psychopathology.

To summarize, studies assessing the influence of gender or age on depressive symptoms has suggested that the prevalence of depression can vary across groups of participants (65, 66). The assessment of depressive symptoms by BDI-II may lead to an overestimation of symptoms among women, as well as to lower reported rates of depression among younger adults. Some items of the BDI-II were amended to match the criteria for DSM-IV depression in 1996, therefore the underlying construct of the inventory encompasses either gender-bounded subjective complaints (e.g., feels sad or empty) or observations made by others (e.g., appears tearful). As a consequence, men with depression may have not be properly identified in clinical practice and remained under-treated. In the same direction, age-related changes in the sexual drive may also be mixed-up with depression-related symptoms, effects of biological transformation and non-disclosure due to social stigma. The current DSM-5 system recognizes heterogeneous phenotypes of depressive symptoms in a dimensional model of severity. Hence, item response approach that accounts for the response probability of the BDI-II in a person-centered approach may better support better the current definition of major depression.

## Limitations

The strength of the current analysis is related to its statistical power and precision of estimates, both derived from the large sample size and gender-balanced distribution of non-clinical participants. However, the interpretation of the results should be considered in light of some limitations. First, despite the sample size, there were a number of response options that were endorsed at low frequency (i.e., <5%). Thus, some parameter estimates may be imprecise. Also, clinical diagnosis of major depression and physical evaluation were not feasible to undertake in such a huge sample.

Second, the non-clinical participants have self-reported the increasing severity level of depressive symptoms through the items of BDI-II. Thus, information bias due to the data collection method might alter the final results. A previous validity study has shown that the self-rated score of the BDI-II was correlated with the assessment of severity in 65.4% of cases of clinically diagnosed DSM-IV major depressive episode (29).

Third, the cross-sectional data of this study would not allow additional inference on factors affecting the different stages of depression severity, covering the onset, the active state, and remission period of depression. A longitudinal design might overcome this deficiency. Fourth, other demographics captured or analysis within-subjects' differences could have also impacted the levels of severity of depressive symptoms, however, they not have been investigated in the current study.

The last concern is related to the generalizability of the findings. Although we have recruited a representative sample of Brazilian college students (the majority in their 20's), the influence of educational level on the BDI-II's psychometric properties was not evaluated in the present study. Therefore, the key findings of this study on the use of the BDI-II should not be extended to the general population or different schooling and age bracket. Further studies should be carried out using a more inclusive sample, with participants from evenly distributed age brackets, diverse educational levels, and recruited from clinical settings.

## Conclusion

Key results of the present investigation have indicated the appropriateness of the BDI-II items as an assessment measure of the severity of depression in a college population. The correct detection of depressive symptoms in student samples may have preventive implications for the community because they are passing by the peak period of onset of depression (6, 67). The item response analysis has indicated that gender and age are variables that hold potential influence on the manifestation of depression.

Although the items “crying” and “interest in sex” have shown noticeable differential item functioning on self-report scales, the impact was trivial. When the BDI-II is applied to a population sample with skewed demographic characteristics or insufficient sample size, the results might be altered likewise. Therefore, it is important that the criteria and instruments used to assess depression can adequately reflect gender- and age-related common symptoms and experiences of depression. The findings of the present investigation provide further support for the validity of using BDI-II for assessing depression in academic contexts.

## Author Contributions

Acquisition of the study was made by AA, Y-PW, LA, and CG. AdS, Y-PW, and CG designed the study. AA, Y-PW, CG, and AdS provided the data. AdS and Y-PW designed the analyses. AdS and GL performed them. AdS, CG, and Y-PW contributed to the interpretation of the data. AdS, GL, CG, and Y-PW wrote the first version of the manuscript. All authors critically revised and approved the final version of the paper.

### Conflict of Interest Statement

The authors declare that the research was conducted in the absence of any commercial or financial relationships that could be construed as a potential conflict of interest.
