# Supplementary material for: Can Gender and Age Impact on Response Pattern of Depressive Symptoms Among College Students? A Differential Item Functioning Analysis
Source: Front Psychiatry. 2019 Feb 12;10:50. doi: 10.3389/fpsyt.2019.00050 (PMC6379252; doi:10.3389/fpsyt.2019.00050)
Supplement: Supplementary file 1 [file Data_Sheet_1.PDF]

Table: BDI-II response proportion (%) for responses categories on 21 items

Item	Overall<br>N=12,677	Men<br>*n* = 5,692	Women<br>*n* = 6,985	Younger<br>*n* = 10,887	Older<br>*n* = 1,790
1. Sadness					
Category 1	81.3	85.7	77.6	80.3	86.9
Category 2	17.5	13.2	20.9	18.3	12.3
Category 3	0.7	0.5	0.8	0.8	0.3
Category 4	0.6	0.5	0.6	0.6	0.5
2- Pessimism					
Category 1	81.0	82.6	79.7	79.9	87.8
Category 2	16.9	15.4	18.2	17.9	11.2
Category 3	1.6	1.6	1.7	1.8	0.7
Category 4	0.5	0.5	0.5	0.5	0.3
3- Past failure					
Category 1	84.3	84.2	84.3	83.7	87.9
Category 2	9.4	10.3	8.7	9.9	6.0
Category 3	5.8	5.0	6.4	5.8	5.4
Category 4	0.5	0.5	0.6	0.5	0.6
4- Loss of pleasure					
Category 1	77.2	80.0	74.9	77.2	77.7
Category 2	19.8	17.5	21.7	19.9	19.1
Category 3	2.4	1.9	2.8	2.4	2.7
Category 4	0.5	0.5	0.6	0.5	0.5
5. Guilty feelings					
Category 1	69.9	72.4	67.9	69.3	73.9
Category 2	28.1	25.9	30.0	28.7	24.8
Category 3	1.4	1.3	1.5	1.4	1.1
Category 4	0.6	0.5	0.6	0.6	0.2
6- Punishment feelings					
Category 1	85.3	85.2	85.4	84.9	87.7
Category 2	10.2	10.9	9.6	10.6	8.0

Category 3	1.3	1.1	1.5	1.4	0.9
Category 4	3.2	2.9	3.4	3.1	3.5
7- Self-dislike					
Category 1	85.5	87.8	83.7	84.5	92.0
Category 2	6.2	6.0	6.4	6.7	3.2
Category 3	6.9	5.4	8.2	7.4	3.9
Category 4	1.3	0.8	1.7	1.4	0.8
8- Self-criticalness					
Category 1	56.0	56.5	55.5	55.2	61.5
Category 2	33.1	33.4	32.9	33.2	32.3
Category 3	9.4	9.2	9.5	10.0	5.6
Category 4	1.5	1.0	2.0	1.7	0.6
9- Suicidal thoughts					
Category 1	93.2	93.6	92.8	92.6	96.5
Category 2	6.1	5.7	6.5	6.6	3.4
Category 3	0.4	0.4	0.3	0.4	0.1
Category 4	0.3	0.3	0.4	0.4	0.1
10- Crying					
Category 1	77.6	88.4	68.9	76.7	83.5
Category 2	12.1	6.4	16.7	12.6	8.8
Category 3	6.5	0.8	11.2	7.0	3.9
Category 4	3.8	4.5	3.1	3.7	3.8
11- Agitation					
Category 1	71.0	74.7	67.9	69.6	79.2
Category 2	25.1	21.9	27.8	26.2	18.5
Category 3	2.2	2.0	2.4	2.4	1.1
Category 4	1.7	1.5	1.9	1.8	1.1
12- Loss of interest					
Category 1	77.0	79.6	74.9	76.1	82.8
Category 2	20.4	18.2	22.3	21.3	15.4

Category 3	1.8	1.6	2.0	1.9	1.2
Category 4	0.7	0.7	0.8	0.7	0.6
13- Indecisiveness					
Category 1	71.6	76.7	67.5	70.4	78.7
Category 2	20.7	18.6	22.4	21.3	17.1
Category 3	2.3	1.8	2.8	2.4	1.5
Category 4	5.4	2.9	7.4	5.8	2.7
14- Worthlessness					
Category 1	88.1	90.4	86.2	87.7	90.6
Category 2	7.0	6.5	7.4	7.1	6.5
Category 3	4.3	2.7	5.6	4.5	2.6
Category 4	0.6	0.5	0.7	0.7	0.2
15- Loss of energy					
Category 1	61.6	66.3	57.7	61.9	59.4
Category 2	33.2	30.1	35.8	32.6	36.4
Category 3	4.5	3.2	5.7	0.47	3.9
Category 4	0.7	0.5	0.9	0.8	0.3
16- Changes in sleep					
Category 1	48.5	51.6	45.9	47.5	54.6
Category 2	38.9	36.7	40.7	39.7	34.2
Category 3	10.4	9.9	10.9	10.7	8.6
Category 4	2.2	1.8	2.5	2.1	2.6
17- Irritability					
Category 1	68.7	73.0	65.1	67.5	75.5
Category 2	26.6	23.6	29.1	27.4	21.7
Category 3	3.6	2.7	4.3	3.8	2.2
Category 4	1.2	0.7	1.5	1.3	0.6
18- Changes in appetite					
Category 1	61.6	69.1	55.4	60.5	68.3
Category 2	31.3	26.2	35.4	32.1	26.3

Category 3	4.9	3.4	6.2	5.2	3.6
Category 4	2.2	1.3	2.9	2.3	1.7
19- Concentration difficulty					
Category 1	65.8	70.0	62.4	65.7	66.3
Category 2	23.4	21.8	24.7	23.3	24.4
Category 3	9.3	7.0	11.2	9.5	8.3
Category 4	1.4	1.2	1.6	1.5	0.9
20- Tiredness or fatigue					
Category 1	59.1	63.6	55.4	58.6	61.8
Category 2	33.2	31.0	34.9	33.4	31.6
Category 3	5.6	4.0	6.9	5.7	5.0
Category 4	2.1	1.4	2.8	2.2	1.6
21. Loss of interest in sex					
Category 1	88.0	91.9	84.8	89.6	78.3
Category 2	9.4	6.9	11.4	8.0	17.7
Category 3	2.0	0.9	2.9	1.9	3.0
Category 4	0.6	0.3	0.9	0.5	1.1
